# PEDF inhibits non-small cell lung cancer proliferation by suppressing autophagy through downregulation of AMPK-ULK1 signaling

**DOI:** 10.3892/or.2022.8434

**Published:** 2022-10-24

**Authors:** Haoran Miao, Hongliang Hui, Huaming Li, Yangui Lin, Dan Li, Min Luo, Bo Jiang, Yiqian Zhang

**Affiliations:** 1 Department of Thoracic Cardiovascular Surgery, The Eighth Affiliated Hospital of Sun Yat-sen University, Shenzhen, Guangdong 518000, P.R. China; 2 Community Health Center, The Eighth Affiliated Hospital of Sun Yat-sen University, Shenzhen, Guangdong 518000, P.R. China

**Keywords:** pigment epithelium-derived factor, autophagy, proliferation, non-small cell lung cancer

## Abstract

Current investigations suggest that pigment epithelial-derived factor (PEDF) can mediate the progression of non-small cell lung cancer (NSCLC) by regulating autophagy. However, the underlying mechanisms associated with autophagy remain poorly elucidated. The aim of the present study was to investigate the association between the PEDF/adenosine 5′-monophosphate-activated protein kinase (AMPK)/Unc-51 like autophagy-activated kinase 1 (ULK1) pathway and autophagy in NSCLC. Intracellular autophagy was evaluated using indicators such as the expression and activation of microtubule-associated protein light chain 3-I (LC3-I), LC3-II and p62, as well as the distribution and number of autophagosomes observed by confocal microscopy. In addition, the activity and proliferative capacity of NSCLC cells under PEDF overexpression was also examined using Cell Counting Kit-8 and lactate dehydrogenase (LDH) assays, and western blotting (WB) of related proteins. The results revealed that PEDF significantly inhibited NSCLC cell proliferation and viability, and increased LDH release and intercellular adhesion. Furthermore, PEDF suppressed the expression and activation of LC-3 and reduced the number and distribution of autophagosomes. The PEDF-induced inhibition of autophagy exhibited a direct association with the suppressed proliferation capacity and cell viability of NSCLC cells. The results of WB showed that NSCLC cells regulated autophagy through the AMPK/ULK1 signaling pathway. PEDF downregulated the AMPK/ULK1 signaling pathway, and AMPK or ULK1 overexpression markedly reduced the inhibitory effect of PEDF on autophagy. In conclusion, PEDF overexpression significantly inhibited the proliferative capacity and cell viability of NSCLC cells, as PEDF exerted an inhibitory function by regulating autophagy in NSCLC cells. Finally, it was demonstrated that autophagy may be suppressed by inhibiting the AMPK/ULK1 signaling pathway, thereby revealing a mechanism of lung cancer progression.

## Introduction

Lung cancer, which remains the leading cause of cancer-related mortality worldwide (1), is divided into small cell lung cancer (SCLC) and non-SCLC (NSCLC). NSCLC accounts for 80–85% of all lung cancer cases (2,3). Although the emergence of novel therapeutic methods has significantly improved the treatment of NSCLC, the prognosis remains poor, with a 5-year survival rate of only 19.3% (4). Thus, there is an urgent need for new therapies.

Autophagy is a highly conserved process in which cellular components are captured and delivered to double-membrane vesicles called autophagosomes, which are subsequently degraded by the lysosomal system (5). Autophagy plays an influential role in tumor development (6). It has been reported that the deletion of Atg7 within tumor cells induces the inhibition of intracellular autophagy, which has been shown in multiple models to impair their growth (7). Chemical or genetic autophagy inhibition delivered systemically blunted tumor growth and invasion (8). Moreover, strategies to inhibit autophagy to enhance reactive oxygen species-induced oxidative damage for synergistic cancer therapy, as well as new autophagy inhibitors, are emerging in clinical trials for antitumor therapy (9,10). Unfortunately, almost all autophagy inhibitors are highly toxic, which limits clinical application (11–13). Therefore, it is particularly urgent to develop new autophagy inhibitors with a low or no toxicity. Pigment epithelial-derived factor (PEDF) is a member of the serine protease superfamily and can regulate proteolytic cascades related to key biological processes, such as blood coagulation, inflammation and angiogenesis (14,15). Studies have shown that PEDF is an effective tumor angiogenesis inhibitor, which could inhibit cancer cell invasion and metastasis to prevent cancer progression (16–18). Zhang *et al* (19) demonstrated that PEDF expression was reduced in NSCLC and was correlated with clinical outcomes. Chen *et al* (20) suggested that the molecular impact of PEDF on lung cancer cells and its clinical implications are significant. PEDF is highly expressed in multiple tissues and is essential for maintaining homeostasis; it is also a key regulator of autophagy and energy metabolism (21,22). Previous studies by the authors revealed that PEDF was involved in the regulation of mitophagy levels and carbohydrate uptake and metabolism in ischemic cardiomyocytes (23–25). However, the effect of PEDF on intracellular autophagy in NSCLC remains unclear.

Therefore, the present study evaluated the effect of PEDF on autophagy status and the related mechanism in lung cancer cells. It was demonstrated that PEDF significantly inhibited lung cancer cell proliferation and viability by suppressing autophagy through the downregulation of the adenosine 5′-monophosphate-activated protein kinase (AMPK)/Unc-51 like autophagy-activated kinase 1 (ULK1) signaling pathway in NSCLC cells.

## Materials and methods

### Materials

Anti-protein kinase C α (PKCα; cat. no. 2056), anti-ULK1 (cat. no. 8054), anti-microtubule-associated protein light chain 3-I (LC3-I; cat. no. 4108), anti-LC3-II (cat. no. 2775), anti-AMPKα (cat. no. 5832), anti-phosphorylated (p)-AMPKα (Thr172, cat. no. 2535) and anti-p-ULK1 (cat. no. 5869) antibodies were purchased from Cell Signaling Technology, Inc. Anti-p-PKCα (cat. no. 07-790) antibody was obtained from MilliporeSigma. Anti-β-actin (cat. no. 66009-1-lg) antibody was purchased from ProteinTech Group, Inc. Bafilomycin A1 (BAF1; cat. no. S1413) was purchased from Selleck Chemicals. Rapamycin (cat. no. HY-10219) was purchased from MedChemExpress.

### Cell culture and reagents

H460 and A549 human NSCLC cell lines were donated by Dr Jingjun Han (Eighth Affiliated Hospital of Sun Yat-sen University, Shenzhen, China) and cell line H1299 (cat. no. SCSP-589) was purchased from the Cell Bank of the Chinese Academy of Sciences (http://www.cellbank.org.cn), HBE135-E6E7 (referred to as HBE hereafter; cat. no. CRL-2741) and hTERT lung fibroblasts (referred to as fibroblasts hereafter; cat. no. CRL-4058) were purchased from ATCC. All the cells were cultured in RPMI-1640 medium with 10% FBS (Cytiva), 100 µg/ml penicillin and 0.1 mg/ml streptomycin. All cells were maintained in a cell culture incubator at 37°C in a humidified atmosphere with 5% CO_2_. All experiments were conducted in the exponential phase of the cells.

### Cell viability and (lactate dehydrogenase) LDH release assay

Cell viability was assessed using Cell Counting Kit-8 (CCK-8) (cat. no. C0038; Beyotime Institute of Biotechnology) assay, according to the manufacturer's instructions. Briefly, NSCLC cell lines, H1299, A549 and H460 were seeded in 96-well plates at a density of 5×10^3^ cells/well for 24 h. The mixture was then treated with CCK-8 reagent and incubated at 37°C for an additional 0.5-3 h. Cell viability was determined by measuring the absorbance at 450 nm using a microplate reader. Each experiment was repeated three times. LDH activity in cell supernatants was detected with LDH Cytotoxicity Assay Kit (cat. no. 4744926001; Roche Diagnostics), according to the manufacturer's instructions.

### Western blotting (WB)

Collected cells were mixed with lysis buffer (500 µl; Shanghai Aladdin Biochemical Technology Co., Ltd.) and placed on ice to lyse for 25 min, followed by centrifugation at 12,000 × g for 15 min at 4°C. A BCA protein Concentration Determination Kit (cat. no. P0012; Beyotime Institute of Biotechnology) was used to determine protein concentration in the supernatant. After mixing the protein sample (5 µl) with 5X sodium dodecyl sulfate loading buffer, the mixture was denaturated by boiling in a water bath for 10 min. The samples (20 µg per lane) were then electrophoresed on a 10% SDS-PAGE (100 V) and transferred to a PVDF membrane on ice (250 mA, 60 min). The PVDF membrane was then sealed with 50 g/l skimmed milk at room temperature for 90 min. Subsequently, PVDF membranes were incubated with primary antibodies overnight at 4°C. The primary antibodies were as follows: Rabbit anti-human ULK1 [cat. no. 8054; 1:1,000; Cell Signaling Technology, Inc. (CST)], LC3-I (cat. no. 4108; 1:500; CST), LC3-II (cat. no. 2775; 1:500; CST), AMPKα (cat. no. 5832; 1:1,000; CST), p-AMPKα (cat. no. 2535; 1:500; CST), PI3K (cat. no. 4249; 1:1,000; CST), MAPK (cat. no. 4695; 1:1,000; CST), PEDF (cat. no. DF6547; 1:1,000; Affinity Biosciences), extracellular signal-regulated protein kinase (ERK; cat. no. 4348; 1:1,000; CST), p-ERK (cat. no. 8544; 1:1,000; CST), p38 (cat. no. 14451; 1:1,000; CST), p-p38 (cat. no. 4511; 1:1,000; CST), mTOR (cat. no. ab2732; 1:1,000; Abcam), TSC (cat. no. ab200728; 1:1,000; Abcam), and p62 (cat. no. ab109012; 1:500; Abcam) primary antibodies as well as mouse anti-human GAPDH (cat. no. ab8245; 1:500; Abcam) and β-actin (cat. no. ab5694; 1:1,000; Abcam) primary antibodies. The membrane was then thoroughly washed three times with PBST (including 0.1% v/v Tween-20) for 5 min each time. Next, PVDF membranes were incubated with HRP-conjugated secondary antibodies (anti-rabbit ab205718 and anti-mouse ab205719; 1:4,000; Abcam) at room temperature for 60 min. Subsequently, the membranes were washed multiple times with PBST and then developed using an enhanced chemiluminescence detection kit (Sigma-Aldrich; Merck KGaA) for imaging. Image Lab V3.0 software (Bio-Rad Laboratories, Inc.) was used to obtain and analyze imaging data. The relative expression of the target protein was expressed as the ratio of GAPDH or β-actin.

### Reverse transcription-quantitative PCR (RT-qPCR)

Total RNA was extracted from NSCLC cells using TRIzol^®^ (Thermo Fisher Scientific, Inc.), according to the manufacturer's instructions (26). Collected NSCLC cells were lysed by 1 ml of TRIzol (Thermo Fisher Scientific, Inc.). Following lysis, total RNA was extracted using the phenol-chloroform method (27). The purity of RNA was determined by UV A260/A280 spectrophotometry (Nanodrop ND2000; Thermo Fisher Scientific, Inc.). cDNA was then obtained by reverse transcription from 1 µg RNA using miScript II RT kit (Qiagen GmbH) and stored at −20°C. RT-qPCR was performed using SYBR Green PCR kit. The reaction system consisted of 10 µl RT-qPCR-mix, 0.5 µl forward primer (human PEDF forward, 5′-ATTCCCGATGAGATCAGCA-3′; and human GAPDH forward, 5′-AGCCACATCGCTCAGACAC-3′), 0.5 µl reverse primer (human PEDF reverse, 5′-CTTAGGGTCCGACATCATGG-3′; and human GAPDH reverse, 5′GCCCAATACGACCAAATCC-3′), 2 µl cDNA and 7 µl double distilled water (ddH_2_O). The reaction protocol was as follows: Initial denaturation at 95°C for 10 min, and 40 cycles at 95°C for 1 min and 60°C for 30 sec. Analysis of relative gene expression data by RT-qPCR was 2^−ΔΔCq^ method (28).

### PEDF lentiviral vector construction and cell transfection assays

Recombinant lentivirus was prepared as previously described (29). Briefly, lentiviral plasmids were transfected into 293SF cells (cat. no. CRL3249; ATCC) with PEI as the transfection reagent. Plasmids were constructed and purified by chromatography using maxiprep plasmid purification kit (Qiagen GmbH). Lentivirus (LVs) was purified by PEG6000. The assay was performed following the manufacturer's instructions (30). Various concentration steps eventually resulted in a titer of 1,010 IU/ml. Cells and LVs were co-cultured to construct stable cell lines with a multiplicity of infection of 10:1.

### Autophagy monitoring assay

A tandem GFP-red fluorescent protein (RFP)-LC3 adenovirus construct obtained from Hanbio Biotechnology Co., Ltd. was used in this study. This tandem GFP-RFP-LC3 construct utilizes the pH difference between acidic and neutral autophagosomes, and the difference in pH sensitivity exhibited by GFP and RFP to monitor the progression from autophagosomes to autolysosomes. In brief, for image-based autophagy analysis, NSCLC cells were infected with tandem GFP-RFP-LC3 adenovirus for 2 h and then were cultured with normal medium for 24 h, and then the cells were treated and imaged for GFP and RFP using fluorescence microscopy. The cells were then observed using a fluorescence microscope (Olympus Corporation) or confocal laser scanning microscope (Olympus Corporation). Image-Pro Plus (Media Cybernetics, Inc.) analyzed the co-localization rates and intensity of LC3/Mito-tracker Red (cat. no. M7512; Invitrogen™; Thermo Fisher Scientific, Inc.).

### Statistical analysis

Data are presented as the mean ± SEM. Data were measured using a two-tailed unpaired Student's t-test for comparison between two groups and one-way ANOVA for multiple comparisons, followed by a Student-Newman-Keuls test. Statistical analysis was performed using PASW Statistics 21 (IBM Corp.). P<0.05 was considered to indicate a statistically significant difference.

## Results

### PEDF inhibits NSCLC proliferation

**Figure 1.**
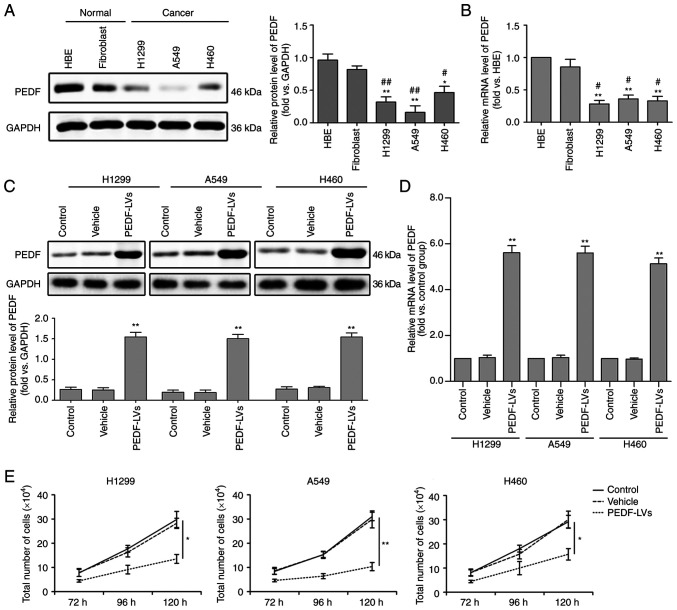
Expression of PEDF in normal and cancer tissues and the effect of PEDF expression on cell proliferation. Three types of NSCLC cell lines H460, A549 and H1299 were assessed, and HBE and fibroblast cell lines were used as normal controls. (A and B) WB was used to determine the expression of PEDF in NSCLC cells. Detection of the mRNA expression of PEDF using RT-qPCR. *P<0.05 and **P<0.01 vs. the HBE group; and ^#^P<0.05 and ^##^P<0.01 vs. the fibroblast group. (C and D) Decreased expression of the protein and mRNA of PEDF was identified by WB and RT-qPCR in H460, A549 and H1299 cell lines transfected with PEDF-overexpressing lentiviral vectors. (E) Following overexpression of PEDF, the number of NSCLC cells was detected at various time-points. *P<0.05 and **P<0.01 vs. the control group. Data are presented as the mean ± standard error of the mean. PEDF, pigment epithelium-derived factor; NSCLC, non-small cell lung cancer; HBE, human bronchial epithelial; WB, western blotting; RT-qPCR, reverse transcription-quantitative PCR; LVs, lentivirus.

To detect the biological function of PEDF on the cellular level in lung cancer, three NSCLC cells (H1229, A549 and H460) and two normal tissue cells (HBE and fibroblasts) were cultured under the same conditions. The expression of PEDF was then detected by WB, and it was found that, compared with normal cells, the expression and mRNA levels of PEDF were significantly decreased in all three NSCLC cell lines (Fig. 1A and B). To confirm this, PEDF overexpression was induced in H1299, A549 and H460 cells and a comparative analysis of the control and vehicle groups was performed. The results revealed increased expression of PEDF protein (Fig. 1C) and mRNA (Fig. 1D) via WB and quantitative PCR in H1229, A549 and H460 cell lines transfected with PEDF-overexpressing lentiviral vectors. In addition, it was determined that PEDF overexpression could significantly reduce the proliferation of NSCLC cells compared with normal cells (Fig. 1E).

**Figure 2.**
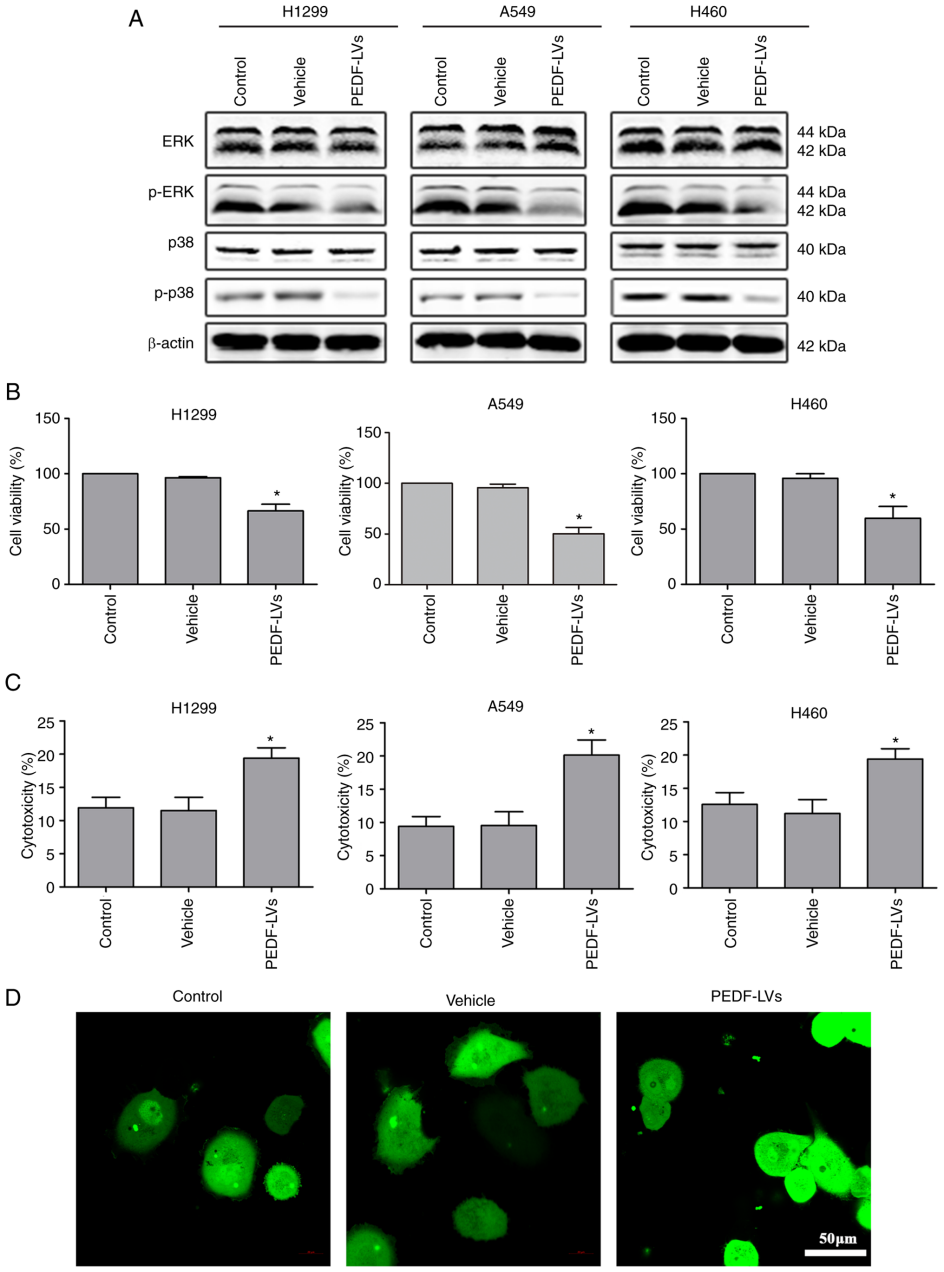
Effects of PEDF overexpression on viability and cytotoxicity of NSCLC cells. (A) Western blotting was used to detect the expression of cyclins (ERK, p-ERK, p38, p-p38) in NSCLC cells, assessing cell proliferation compared with the control and vehicle group. (B) A CCK-8 assay was used to assess the inhibitory effects of PEDF-LVs on the viability of NSCLC cells. (C) An LDH assay was used to assess the cytotoxicity of PEDF in NSCLC cells. (D) A549 cells were analyzed for cell-cell adhesion by confocal microscopy (green, cytoplasm); scale bar, 50 µm. *P<0.05 vs. the control group. Data are presented as the mean ± standard error of the mean. PEDF, pigment epithelium-derived factor; NSCLC, non-small cell lung cancer; ERK, extracellular signal-regulated kinase; p-, phosphorylated; CCK-8, Cell Counting Kit-8; LDH, lactate dehydrogenase; LVs, lentivirus.

In addition, the expression of ERK, p-ERK, p38 and p-p38 was examined. The results revealed that the expression of p-ERK and p-p38 was decreased, which indicated that the proliferation of NSCLC cells was inhibited (Fig. 2A). Similarly, the results of the CCK-8 and LDH assays showed that PEDF overexpression resulted in a distinct decrease in cell viability and a notable increase in cytotoxicity (Fig. 2B and C). The confocal microscopy results showed that PEDF overexpression clearly increased the aggregation of cancer cells (Fig. 2D). Collectively, it was demonstrated that PEDF expression inhibited the proliferation of NSCLC cells.

### PEDF reduces NSCLC proliferative activity by negatively regulating autophagy

**Figure 3.**
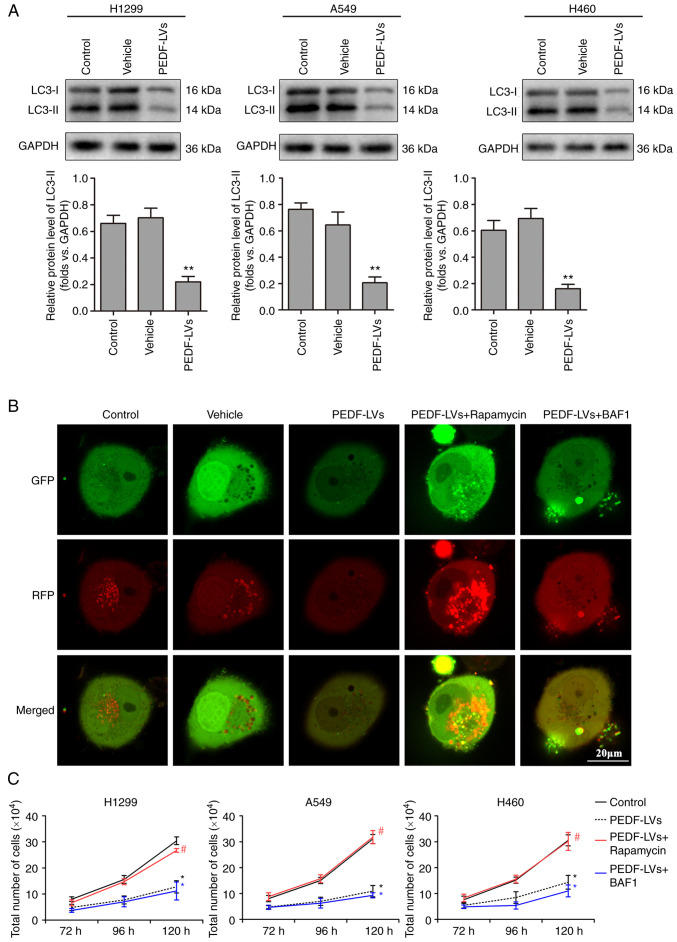
Relative expression levels of LC3-I, LC3-II and autophagosomes in NSCLC cells. (A) The cells were transfected with PEDF-LVs and vehicle before determination of LC3-I and LC3-II expression by western blotting; n=4. (B) Cells were treated with tandem GFP-RFP-LC3 adenovirus, and confocal microscopy was used to observe the morphological changes of autophagosomes; n=4. (C) The number of NSCLC cells was detected at different time-points and the effects of autophagy activators and inhibitors on the function of PEDF in inhibiting proliferation was determined; n=4. *P<0.05 and **P<0.01 vs. the control group; and ^#^P<0.05 vs. the PEDF-LVs group. Data are presented as the mean ± standard error of the mean. LC3-I, microtubule-associated protein light chain 3-I; NSCLC, non-small cell lung cancer; PEDF, pigment epithelium-derived factor; LV, lentivirus; GFP, green fluorescent protein; RFP, red fluorescent protein; BAF1, bafilomycin A1.

Abnormal autophagy is closely associated with the presence of tumors, neurodegenerative diseases and metabolic and immune diseases (31). LC3-I is activated by APG7L/ATG7, translocates with ATG3 and is coupled with fatty acyl ethanolamine (PE) to form the membrane-bound form LC3-II, which can attach to the membrane of autophagosomes and is the structural protein of autophagosomes (32). LC3-II is often considered a marker of autophagosomes (33). In the previous experiment, it was demonstrated that PEDF overexpression markedly inhibited the proliferation of NSCLC cells, which was hypothesized may be influenced by autophagy. The expression of LC3 was therefore examined. The results revealed that PEDF overexpression downregulated the autophagy marker LC3 compared with the vehicle group, indicating that PEDF negatively regulates autophagy in NSCLC cells (Fig. 3A). Mitochondrial autophagy was detected using a tandem GFP-RFP-LC3 adenovirus plasmid, which represents autophagosome formation. When autophagosomes fuse with lysosomes to form autolysosomes, GFP molecules are degraded from the tandem proteins, but RFP-LC3 remains punctate (34). As shown in Fig. 3B, cells with PEDF overexpression transfected with the GFP-RFP-LC3 plasmid exhibited low LC3 expression. However, autophagy activator rapamycin reversed this outcome, whereas autophagy inhibitor Myb-like DNA-binding protein BAS1 had no impact on the inhibition of autophagy caused by PEDF overexpression (Fig. 3B). These results indicated that PEDF overexpression affected the expression of autophagic proteins and decreased the formation of phagosomes. Furthermore, it was explored whether the inhibition of NSCLC proliferation by PEDF is related to its negative regulation of autophagy. Following PEDF overexpression, NSCLC cells were treated with rapamycin or BAF1 in culture for 120 h, respectively. The autophagy activator rapamycin reversed the inhibitory effect of PEDF on the proliferative activity of NSCLC cells, whereas the autophagy inhibitor BAF1 had no effect (Fig. 3C). The aforementioned results indicated that PEDF inhibits the proliferative activity of NSCLC cells by reducing autophagy.

### ULK1 is critical for PEDF-induced autophagy in NSCLC

**Figure 4.**
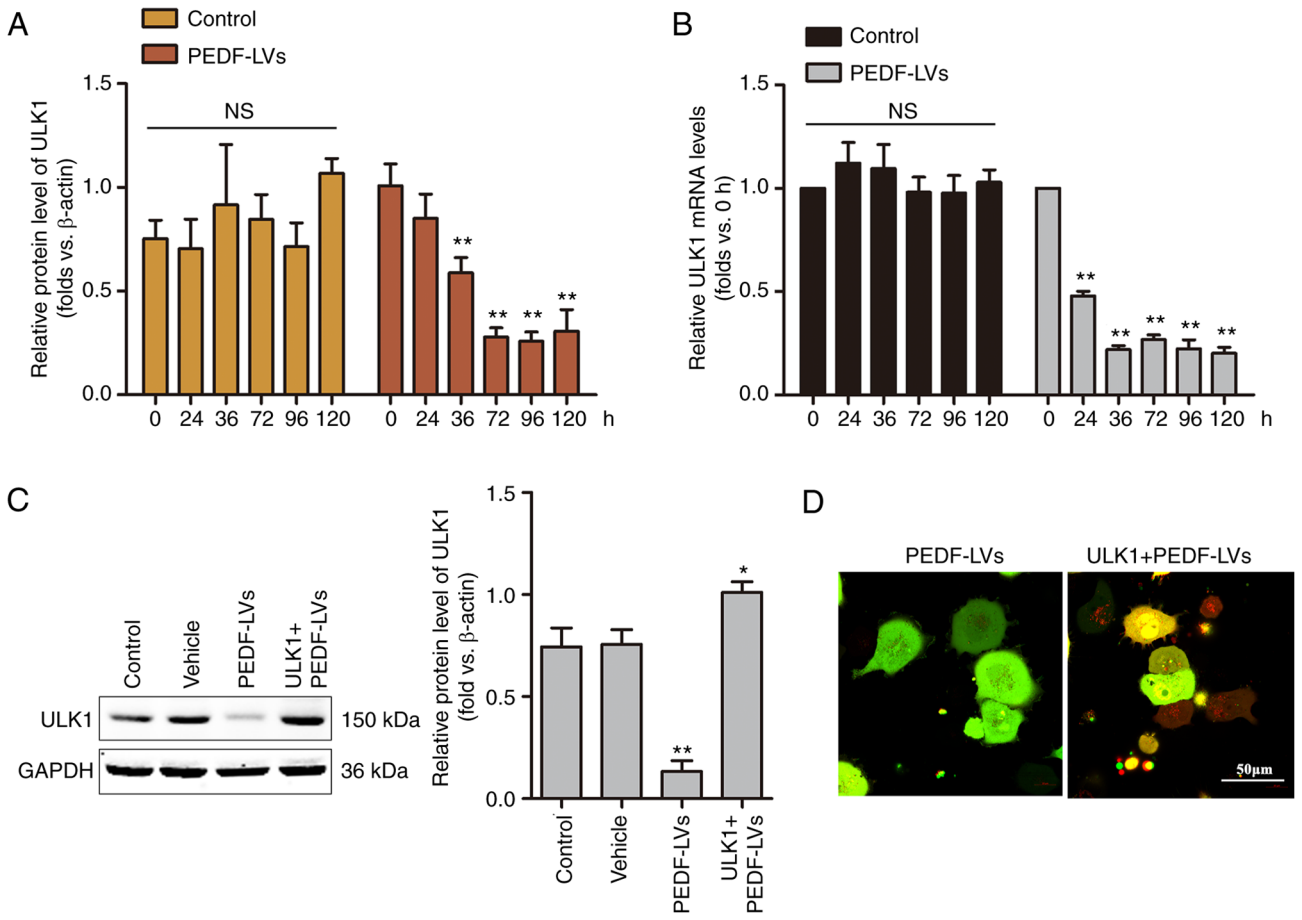
ULK1 is involved in PEDF-induced autophagy. (A) The expression of ULK1 protein at different incubation times was detected by WB, and the differences between the PEDF overexpression group and the control group were compared and analyzed; n=4. **P<0.01 vs. the 0-h group. (B) The expression of ULK1 mRNA at different incubation times was detected by reverse transcription-quantitative PCR; n=4. **P<0.01 vs. the 0-h group. (C) WB determination of ULK1 protein levels in cells under the simultaneous intervention of ULK1- and PEDF-overexpressing lentivirus; n=4. *P<0.05 and **P<0.01 vs. the control group. (D) Cells were treated with tandem GFP-RFP-LC3 adenovirus, and confocal microscopy was used to observe the morphological changes of autophagosomes in two groups overexpressing PEDF and ULK1 + PEDF; n=4. ULK1, Unc-51 like autophagy-activated kinase 1; PEDF, pigment epithelium-derived factor; WB, western blotting; GFP, green fluorescent protein; RFP, red fluorescent protein; LC3, microtubule-associated protein light chain 3; LV, lentivirus.

Among the molecular mechanisms of macroautophagy, ULK1, which is homologous to the yeast autophagy gene Atg1, is a key autophagy-initiating kinase that integrates cellular nutrients and energy and regulates the induction of autophagy (35). To investigate the role of ULK1 on PEDF-induced autophagy, a time-course analysis of the mRNA and protein expression of ULK1 was performed using RT-qPCR and WB, respectively. As demonstrated in Fig. 4A and B, the mRNA and protein levels of ULK1 were decreased within 36 h and remained low to 120 h. The results demonstrated that the overexpression of PEDF induced a significant decrease in the protein expression of ULK1 in NSCLC cells, suggesting that PEDF plays a key role in regulating autophagy through the involvement ULK1 signaling. To verify the hypothesis that ULK1 is involved in mediating PEDF-induced autophagy in NSCLC cells, a ULK1-overexpressing lentivirus was constructed, and the effect was confirmed by WB (Fig. 4C). In the present study, following the overexpression of ULK1 and co-expression of ULK1 with PEDF in NSCLC cells, confocal microscopy results revealed a punctiform distribution of autophagosomes in cells overexpressing PEDF, while autophagosomes in cells co-expressing PEDF + ULK1 showed a diffuse distribution with a noticeable increase in red spots (Fig. 4D). The results demonstrated that PEDF overexpression suppressed ULK1 expression in NSCLC cells, indicating that PEDF plays a key role in regulating autophagy through the involvement of ULK1 signaling.

### Effects of PEDF on the expression of genes downstream of the AMPK pathway in NSCLC cells

**Figure 5.**
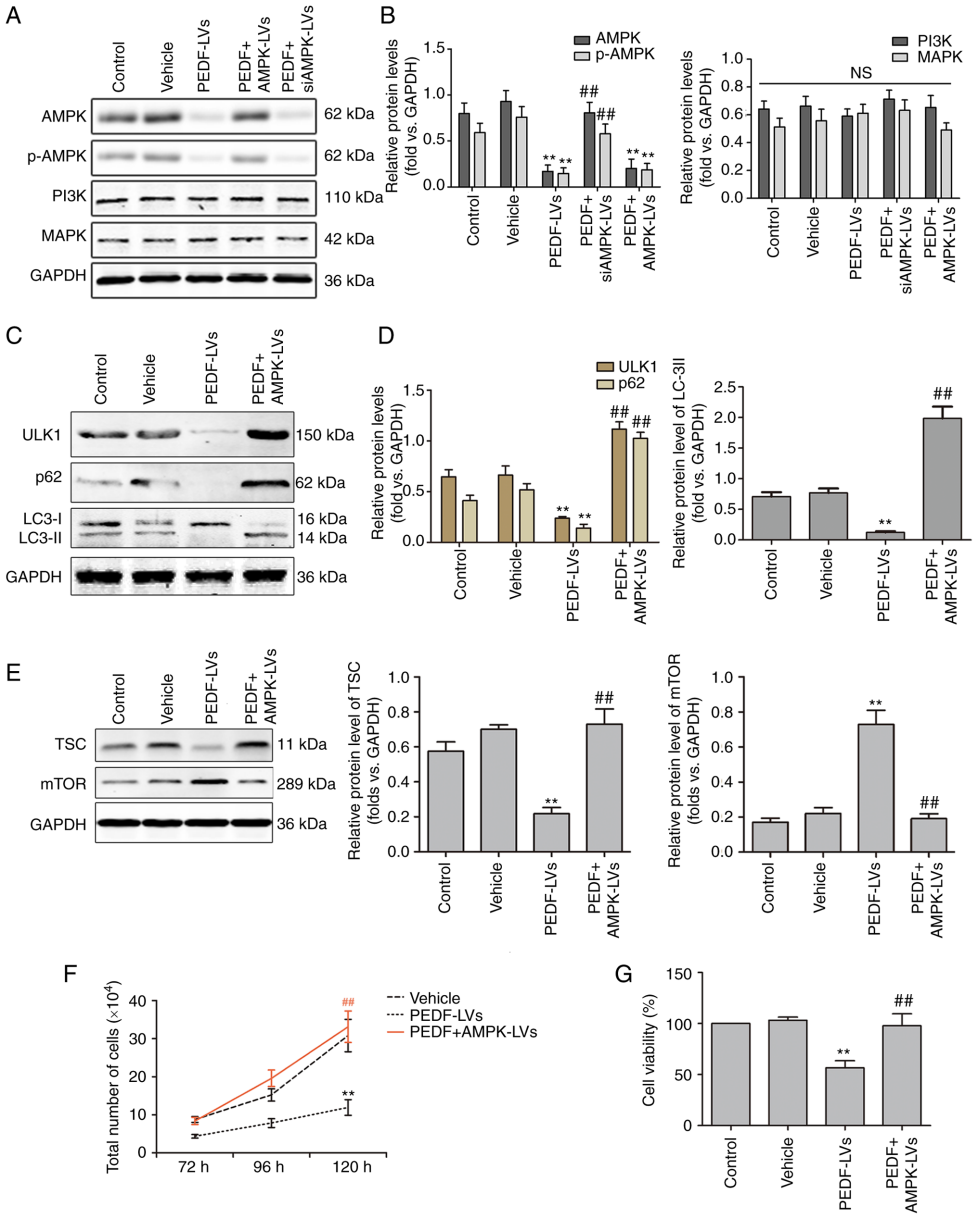
PEDF regulates autophagy via the AMPK/ULK1 signaling pathway in NSCLC cells. (A and B) Western blotting was used to determine the expression of AMPK, p-AMPK, PI3K, and MAPK. PEDF downregulates total and p-AMPK, which could be reversed by AMPK-overexpressing lentiviral delivery to cells; n=4. (C and D) Protein levels of ULK1, p62, LC3-I, and LC3-II in NSCLC cells treated with PEDF-LVs with or without AMPK overexpression lentivirus; n=4. (E) Protein levels of TSC and mTOR treated with PEDF-LVs with or without AMPK overexpression lentivirus; n=4. (F) The number of cells cultured for 72–120 h with PEDF-LVs with or without AMPK-LVs intervention was assessed; n=4. (G) Cell viability assay; n=3. **P<0.01 vs. the control group; and ^##^P<0.01 vs. the PEDF-LVs group. PEDF, pigment epithelium-derived factor; AMPK, adenosine 5′-monophosphate-activated protein kinase; ULK1, Unc-51 like autophagy-activated kinase 1; NSCLC, non-small cell lung cancer; p-, phosphorylated; PKCα, anti-protein kinase C α; LC3, microtubule-associated protein light chain 3; LV, lentivirus.

The ULK1-mediated upstream regulation of autophagy consists of three main signaling pathways, namely the AMPK, PI3K/Akt and MAPK/ERK1/2 signaling pathways (36–38). AMPK is a known upstream regulator of ULK1 that phosphorylates and activates ULK1 at multiple sites in a cross-talk manner (37). The results of WB revealed that the overexpression of PEDF decreased the protein expression of AMPK compared with the empty vector control or DMSO control group (Fig. 5A and B). However, it had no effect on the protein expression of PI3K and MAPK. The effect of PEDF on PI3K and MAPK is dual, where PEDF inhibits them under hypoxia but has no effect under normoxia (39–42). Therefore, it was hypothesized that PEDF inhibits autophagy by downregulating the AMPK signaling pathway. Subsequently, the association between PEDF and the autophagy-associated AMPK/ULK1 signaling pathway was investigated. The data obtained using WB revealed that PEDF reduced ULK1 expression through inhibiting AMPK, and AMPK overexpression reversed the effects of PEDF (Fig. 5C and D). The expression of mTOR, which is a negative regulator of ULK1, was also assessed. AMPK was able to inhibit mTOR directly and indirectly by activating TSC (43). As shown in Fig. 5E, PEDF markedly supressed TSC expression and increased mTOR expression, while overexpression of AMPK reversed these effects. In addition, PEDF overexpression significantly decreased p62 and LC3-II expression, but AMPK overexpression markedly increased their expression (Fig. 5C and D). Furthermore, PEDF significantly inhibited NSCLC cell proliferation and viability, while AMPK overexpression reversed this effect (Fig. 5F and G). These results indicated that PEDF regulates the proliferative activity and autophagy of NSCLC cells through the AMPK/ULK1 pathway.

## Discussion

In the present study, it was found that PEDF inhibited autophagy in lung cancer cell lines by reducing the expression and activation of AMPK. Of note, in a previous study (23), it was found that PEDF also inhibited AMPK levels in hypoxic cardiomyocytes, whereas autophagy was markedly increased, which was inconsistent with the findings of the present study obtained from lung cancer cell lines. In another study, the mechanism through which PEDF can induce a significant increase and activation of PKCα in hypoxic cardiomyocytes, thereby displacing AMPK to activate the ULK1 signaling pathway and inducing higher levels of autophagy, was elucidated (23). By contrast, PEDF had no effect on the expression of PKCα in lung cancer cell lines (data not shown). This may be the reason why PEDF inhibits autophagy rather than activates it in lung cancer cell lines. It was then demonstrated that PEDF exerts an inhibitory function by suppressing intracellular autophagy in NSCLC cells. Finally, the signaling pathways involved in autophagy were explored, and it was found that PEDF reduces the occurrence of autophagy by blocking the AMPK/ULK1 signaling pathway.

According to lung cancer statistics, NSCLC accounts for ~85% of reported lung cancer cases, and nearly 80% of patients with NSCLC are diagnosed at an advanced stage (2). Patients with advanced NSCLC often lose the opportunity of surgery due to extensive metastasis. Radiotherapy and chemotherapy can easily induce drug resistance in tumor cells, resulting in a very poor prognosis for patients. In recent years, the role of angiogenesis in tumor development, growth and metastasis has been a research hotspot. Of note, PEDF molecules have been revealed to inhibit various malignant phenotypes of NSCLC and have emerged as potential tumor therapeutic targets. In a study by Zhang *et al*, PEDF was reduced at both the protein and mRNA levels in NSCLC tumors compared with normal lung tissue. This decrease was associated with an increase in microvessel density in tumors. The increased microvessel density in tumors was associated with a significant correlation between TNM stage, tumor size and overall survival. This suggests that PEDF is an important factor in the development of NSCLC and may have a prognostic value for patients with NSCLC (19). Chen *et al* (20) also demonstrated that reduced levels of PEDF in lung cancer tissue was significantly correlated with lymph node metastasis and poor overall prognosis in patients with lung cancer. PEDF inhibited the growth and motility of lung cancer cells and was significantly correlated with the clinical outcome of patients. In the present study, it was found that PEDF overexpression in NSCLC significantly decreased autophagy marker proteins p62 and ULK1, and inhibited the proliferative capacity and viability of NSCLC cells. AMPK is a key molecule that regulates biological energy metabolism and autophagy. Current studies have suggested that AMPK plays an essential role in regulating cellular energy homeostasis in eukaryotic cells, including tumor cells, and restoring the normal function of the liver and other tissues in diabetic patients (44). Once activated, AMPK is involved in the regulation of four major types of metabolism in mammals: Protein metabolism, lipid metabolism, carbohydrate metabolism, as well as autophagy and mitochondrial homeostasis (45). Activated AMPK induces the phosphorylation of ULK1, leading to the activation of autophagy. Thus, the question is raised of whether there is an association between PEDF and AMPK. A study by Qiu *et al* (25) found that PEDF promoted proteasomal degradation of AMPK and subsequently reduced ATP production. Yang *et al* (46) reported that metformin inhibited PEDF expression and secretion in adipocytes and hepatocytes by promoting AMPK phosphorylation. However, as no correlation between lung cancer-derived AMPK functional activity and PEDF expression has been reported, further studies are required to fully elucidate this mechanism. The results of the present study demonstrated that PEDF clearly downregulated AMPK expression, indicating that PEDF suppressed the AMPK-related signaling pathways. The present study also explored the role of the downstream factor AMPK in autophagy and the results revealed that PEDF reduced ULK1 expression through AMPK, thus suggesting that PEDF blocked ULK1-induced autophagy. Of note, in the present study it was found that rapamycin promoted the growth of NSCLC cells, but some previous studies have revealed the opposite results. Chen *et al* (47) showed that rapamycin greatly enhanced dasatinib-induced cell growth inhibition and cell cycle G1 arrest in human lung adenocarcinoma A549 cells, without affecting apoptosis. Sun *et al* (48) demonstrated that a combination of rapamycin and trametinib could more effectively inhibit NSCLC cell viability and proliferation. The reason for this discrepancy between the results of the present study and those of the aforementioned two studies may be that rapamycin used alone to treat cells mainly activates the autophagy pathway, while synergistic action with other anticancer drugs affects other pathways, thus leading to different results.

**Figure 6.**
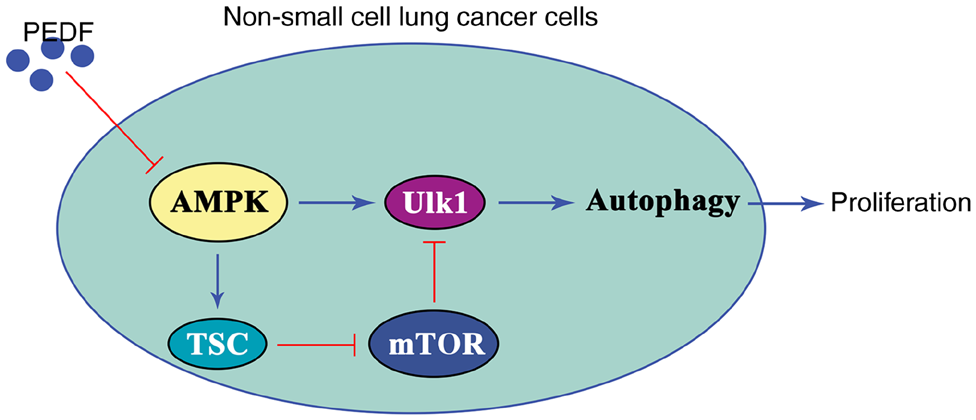
PEDF inhibits the expression and activation of AMPK, leading to suppression of ULK1 directly and indirectly via the TSC-mTOR pathway. Ultimately, PEDF induces autophagy inhibition and suppresses cell proliferation in non-small cell lung cancer cells. PEDF, pigment epithelium-derived factor; AMPK, adenosine 5′-monophosphate-activated protein kinase; ULK1, Unc-51 like autophagy-activated kinase 1.

In conclusion, the results of the present study suggested that PEDF exerts an anticancer effect by inhibiting autophagy induced by the AMPK/ULK1 signaling pathway in lung cancer cells. Specifically, PEDF inhibited the expression and activation of AMPK, leading to inactivation of ULK1 and ultimately inducing autophagy inhibition and suppressed cell proliferation in NSCLC cells (Fig. 6). The overexpression of PEDF inhibited the proliferation and viability of NSCLC cells and significantly reduced the metastatic potential of NSCLC cells. However, the results of the present study only provide a reference point rather than the final conclusion. The reason for this is that the regulation of autophagy relies on a set of complex and complete signaling networks, and its mechanism still needs to be verified by a large number of experiments. In future studies, animal experiments should be performed to further validate the results of the present study at the *in vivo* level, and bioinformatics analysis is also required to elucidate the relevant mechanisms.

## Data Availability

The datasets used and analyzed during the current study are available from the corresponding author on reasonable request.
